# Gut Microbiota Changes Following Aerobic Exercise in Malnourished Octogenarians: An Assessor-Blinded Intervention Study Stratified by Nutritional Status

**DOI:** 10.3390/nu18101627

**Published:** 2026-05-20

**Authors:** Huizhi Yang, Jiahao Li, Shuangfeng Ren, Xinyu Chai, Jiali Lu, Huiping Yan, Yifan Lu

**Affiliations:** 1 School of Sports Medicine and Rehabilitation, Beijing Sport University, Beijing 100084, China; huizhi_yangmm@163.com (H.Y.); leejhfighting@163.com (J.L.); rehabrsf@163.com (S.R.); 13285369937@163.com (X.C.); inicole0322@163.com (J.L.); 2 Key Laboratory of Sports and Physical Fitness of the Ministry of Education, Beijing Sport University, Beijing 100084, China

**Keywords:** aerobic exercise, malnutrition, elderly, intestinal flora

## Abstract

Background/Objectives: Global population aging is associated with a rising prevalence of malnutrition among adults aged ≥80 years. Gut dysbiosis is linked to immune decline and impaired nutrient absorption, and aerobic exercise may enhance microbial diversity. This study investigated gut microbiota changes after a 12-week aerobic exercise intervention in octogenarians stratified by nutritional status. Methods: A total of 129 nursing home residents (≥80 years) were classified via the Mini Nutritional Assessment Short-Form (MNA-SF) into a healthy group (HG, MNA-SF ≥ 11) and a malnourished group (MG, MNA-SF < 11). Both groups underwent a 12-week brisk walking intervention (three sessions/week, 1 h/session, 40–60% heart rate reserve). Fecal samples were collected at baseline and post-intervention and were analyzed via shotgun metagenomic sequencing. Results: A total of 36 participants completed the intervention (HG = 17, MG = 19). Within-group baseline-to-post-intervention analysis showed no significant changes in alpha or beta diversity in the MG. However, post-intervention between-group comparison revealed higher microbial richness and diversity in the MG vs. the HG, with enrichment of taxa including *Faecalibacterium prausnitzii* and *Streptococcus salivarius*. Functional analysis revealed significant enhancements in metabolic pathways related to amino acid biosynthesis, protein synthesis, and quorum sensing in the MG. In contrast, the HG showed limited shifts in microbial diversity but an increase in species involved in carbohydrate metabolism. Conclusions: After 12 weeks, the malnourished group showed higher post-intervention microbial richness and diversity than the healthy group, with differences in taxonomic and predicted functional profiles. Without a non-intervention control group, the microbiota differences observed during the 12-week aerobic exercise period can only be considered observational associations, not causal. Additionally, the high dropout rate (72.1%) limits the generalizability of the findings. Clinical trial registration: The Chinese Clinical Trial Registry on 19 October 2022 (ChiCTR2200064801).

## 1. Introduction

The trend of population aging highlights health issues in those aged ≥80 years [1,2]. Due to declining physical function and the prevalence of comorbidities, this group represents the highest-risk population for malnutrition and its associated health risks [3,4,5]. Malnutrition not only compromises immune function and increases infection risk but also severely impacts the quality of life for the elderly [6,7,8]. As a key regulator of nutrient absorption and immune defense, gut microbiota imbalance is closely associated with various metabolic disorders and chronic disease risks [9,10].

Interventions for malnutrition include physical activity and exercise, dietary education and behavioral interventions, and social support and care, among others [11,12]. Recent studies suggest that aerobic exercise, particularly moderate-intensity activities such as walking, can positively influence the gut microbiota of older adults [11,13,14,15]. Through exercise, the diversity of gut microbiota can be improved, which may help enhance nutrient absorption, strengthen the immune system, and reduce the risk of diseases associated with aging [13]. Aerobic exercise has been shown to modulate the microbial environment of the gut, fostering a healthier microbiome that can improve health outcomes in elderly individuals, especially those suffering from malnutrition [16,17].

Given the growing evidence of the importance of gut microbiota in aging and the potential benefits of exercise, it is crucial to explore effective interventions that can enhance the health of elderly individuals. This study examines the effects of 12 weeks of aerobic exercise on the gut microbiota of individuals aged ≥80, aiming to provide insights into non-pharmacological strategies to improve nutritional status and overall health.

## 2. Materials and Methods

The study used the CONSORT reporting guidelines [18].

### 2.1. Design

**Figure 1:**
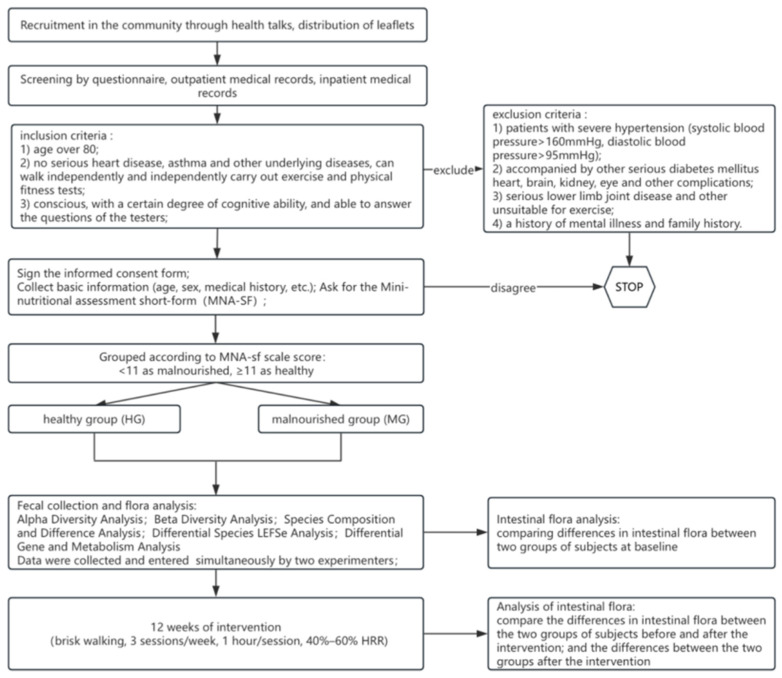
Detailed overview of the research program.

This study was an assessor-blinded intervention study stratified by nutritional status. Participants were stratified into the healthy group (HG) and the malnourished group (MG) according to baseline MNA-SF scores, and both groups underwent the same 12-week aerobic exercise intervention. The gut microbiota was assessed at baseline and again after the 12-week intervention. Analyses included baseline between-group comparisons (HG vs. MG), within-group baseline-to-post-intervention comparisons in each group, and post-intervention between-group comparisons. The detailed procedure is shown in Figure 1.

### 2.2. Participant Recruitment

Participant recruitment was conducted from July 2021 to August 2021. All participants were enrolled from a community-based nursing facility in Langfang City, Hebei Province, China. Subjects were screened based on questionnaires, outpatient medical records, and hospitalization records. The inclusion criteria were (1) age 80 years or older; (2) no serious heart disease, asthma, or other underlying conditions; the ability to walk independently and perform exercise and physical fitness tests; and (3) clear consciousness, sufficient cognitive ability, and the capacity to respond to questions from the testers. Exclusion criteria included patients with severe hypertension (SBP > 160 mmHg, DBP > 95 mmHg); serious diabetic complications affecting the heart, brain, kidneys, or eyes; severe lower limb joint diseases or other conditions making exercise unsuitable; and patients with a history of mental illness or a family history of mental disorders. The intervention was conducted during a period of relative pandemic stability in China, with minimal restrictions and no reported infections among participants or staff. Recruitment criteria, setting, assessment tools, and intervention procedures were kept consistent across both groups.

A total of 129 elderly participants from senior centers were included in the study and divided into two groups according to MNA-SF scale scores. Scores < 11 were classified as malnourished, while scores ≥ 11 were classified as healthy. Each group consisted of 69 (MG) and 60 (HG) participants, respectively. The basic demographic information of the participants is shown in Table 1. The MNA-SF is shown in Table 2.

According to the standard MNA-SF classification, scores of 12–14 indicate normal nutritional status, 8–11 indicate a risk of malnutrition, and 0–7 indicate malnutrition. In this study, a cutoff of 11 was used as a pragmatic threshold to dichotomize participants into relatively higher versus lower nutritional status groups.

#### Sample Size

An a priori sample size calculation was performed using G*Power software (version 3.1.9.7). Based on a similar exercise intervention study, the parameters were set as follows: power (1 − β) = 0.8, α error probability = 0.05, and effect size f = 0.25. Using an F-test for between-group differences with 2 groups and 2 measurements, the calculation yielded a total sample size of 128 participants. We aimed to recruit this target and successfully enrolled 129 participants.

### 2.3. Participant Flow Diagram

**Figure 2:**
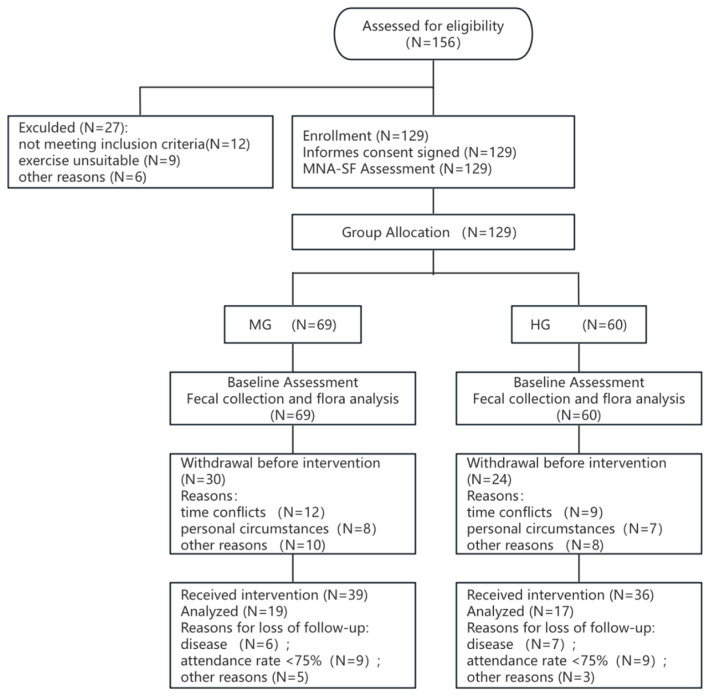
Participant flow diagram of participant progress through the phases of the trial.

Figure 2 presents the participant flow diagram, detailing recruitment, allocation, intervention, follow-up, and reasons for dropout.

### 2.4. Exercise Intervention

The aerobic exercise intervention was delivered as supervised brisk walking. All participants underwent a personalized 12-week brisk walking program based on their heart rate reserve (HRR), with an intensity of 40–60% HRR, a frequency of three sessions per week, and a duration of one hour per session. The intervention was supervised by research staff, and exercise intensity was monitored in real time using sports watches to ensure target intensity and participant safety.

Participant safety was ensured through continuous monitoring by laboratory personnel and on-site medical staff. Participants were instructed to immediately cease exercise and rest if discomfort occurred. Medical personnel then conducted examinations and evaluations and provided necessary treatment, with urgent referrals for serious symptoms.

This study employed an assessor-blinded design. While participants and intervention providers were aware of group assignments, outcome assessors remained blinded to allocation throughout the trial to minimize assessment bias.

Participants were asked to maintain their usual diet, medication regimen, and daily living habits during the intervention period. However, dietary intake, antibiotic or probiotic use, and other medication-related factors were not systematically recorded or controlled.

### 2.5. Data Collection and Analysis

#### 2.5.1. Data Collection

- Questionnaires: (1) Basic information: Age, sex, medical history, and physical activity; (2) Nutritional status: MNA-SF, with scores < 11 indicating malnutrition.
- Anthropometric Measurements: (1) Height: Measured using a stadiometer (precision: 0.01 cm); (2) Body composition: Weight, BMI, and body fat percentage assessed via an InBody230 bioimpedance analyzer (contraindications: cardiac pacemakers; electrodes disinfected with alcohol); (3) Physical function: Tests included 6 min walk, 30 s chair stand, 30 s arm curl, sit-and-reach, handgrip strength, and 8-foot up-and-go. Additional body-composition parameters obtained by InBody230, including appendicular skeletal muscle mass, appendicular skeletal muscle index (ASMI), and body fat percentage, were collected at baseline as complementary objective indicators of nutritional status.
- Fecal samples were self-collected from the first morning stool on the sampling day using OMNIgene GUT kits with room-temperature stabilizers. Swab samples were immersed in stabilizer, the shafts were removed, and samples were stored and transported at ambient temperature before long-term storage at −80 °C. Non-compliant samples were excluded, and all eligible samples underwent shotgun metagenomic sequencing.

#### 2.5.2. Data Analysis

Data analyses were performed in R (version 4.3.1). For continuous variables, data distribution was assessed before statistical testing. Variables meeting the assumption of normality were compared using t-tests, whereas non-normally distributed variables were analyzed using appropriate non-parametric tests. Baseline bioimpedance-derived body-composition indicators were compared between groups, and their associations with baseline MNA-SF scores were assessed using group-comparison tests according to data distribution and Spearman correlation analysis. Species-level taxonomic profiling was performed using MetaPhlAn2. Alpha diversity indices were calculated using the vegan and picante packages and compared according to data distribution. Beta diversity was assessed using Bray–Curtis distance, visualized by principal coordinates analysis (PCoA), and tested by permutational multivariate analysis of variance (PERMANOVA) with 999 permutations using the adonis2 function in the vegan package. Differential taxonomic features were identified using LEfSe implemented in the microeco package based on relative-abundance profiles after normalization. The LEfSe workflow included Kruskal–Wallis testing, Wilcoxon rank-sum testing, and linear discriminant analysis (LDA). An LDA threshold of >2.0 and significance thresholds of *p* < 0.05 or *p* < 0.01 were used as specified. Because LEfSe-derived taxonomic findings were presented separately by comparison and taxonomic rank, Benjamini–Hochberg FDR correction was additionally applied within each reported comparison and stratified by taxonomic rank. KEGG enrichment analysis was performed using clusterProfiler, with Benjamini–Hochberg correction applied to enrichment *p*-values. Given the modest final sample size, the performance of multiple pairwise comparisons, and the descriptive nature of the microbiome analyses, LEfSe-derived taxonomic signals and pathway enrichment results were treated as exploratory and hypothesis-generating. Accordingly, these findings were interpreted as supportive results rather than primary confirmatory evidence.

In addition, an attrition analysis was performed to compare baseline characteristics between completers and non-completers and to examine between-group differences in attrition patterns between the HG and MG. Categorical variables were compared using Fisher’s exact test or chi-square tests, as appropriate, and continuous variables were compared using independent-samples t-tests. All tests were two-sided, with *p* < 0.05 considered statistically significant. A conservative sensitivity analysis was conducted for available continuous clinical and nutritional outcomes (body weight, BMI, and MNA-SF) using baseline observation carried forward (BOCF) for missing post-intervention values. Comparable imputation-based analyses were not feasible for sequencing-based microbiome outcomes because many dropouts did not provide post-intervention stool samples.

Functional pathway analyses were based on shotgun metagenomic annotation and therefore reflect inferred microbial functional potential rather than direct measurements of microbial metabolites or host physiological responses.

## 3. Results

A total of 129 adults aged 80 years and above were enrolled in the study. After the 12-week intervention and exclusion of participants due to epidemic-related restrictions, absence from the facility, illness, or attendance below 75%, 36 participants completed the intervention (MG = 19, HG = 17). The baseline characteristics of the subjects who completed the intervention are shown in Table 3.

To assess the potential impact of attrition, additional attrition analyses were performed (Table S1: Attrition analysis). Although the overall attrition rate was high (72.1%), no statistically detectable differences were observed between completers and non-completers in the available baseline variables, and no statistically detectable between-group differences were found in attrition rate, dropout stage, or dropout reasons. However, given the substantial attrition, limited statistical power, and the per-protocol nature of the primary microbiome analyses, substantial attrition bias cannot be excluded. A conservative sensitivity analysis was further conducted for available continuous clinical and nutritional outcomes (Table S2: Sensitivity analysis using baseline observation carried forward (BOCF) for available continuous clinical/nutritional outcomes). The overall pattern of BMI and body weight findings was unchanged under BOCF, and the direction of MNA-SF changes remained consistent, suggesting that the available non-sequencing outcomes were not materially altered under a conservative missing-data assumption.

Baseline bioimpedance-derived body-composition indicators supported the MNA-SF-based nutritional grouping (Table S3: Baseline objective body-composition indicators according to nutritional status grouping and their correlations with baseline MNA-SF scores). Although ASM, ASMI, and body fat percentage did not differ significantly between the HG and MG at baseline, MNA-SF scores were positively correlated with ASM and ASMI, suggesting consistency between the grouping and muscle-related indicators. The small baseline difference in BMI between the two groups was consistent with the supplementary analysis, which also showed no significant between-group differences in ASM, ASMI, or body fat percentage at baseline (Table S3).

Table 4 summarizes the primary diversity findings of the study. The LEfSe-derived taxonomic differences and functional pathway enrichment results are presented as exploratory, hypothesis-generating analyses intended to support descriptive interpretation rather than to serve as primary evidence.

### 3.1. Differences in Intestinal Flora Between the Two Groups at Baseline

#### 3.1.1. Alpha and Beta Diversity Analysis

At baseline, no significant differences were observed in either alpha diversity (as measured by Chao1, Shannon, Richness and Simpson) or beta diversity structure between the MG and the HG (all *p* > 0.05), indicating similar overall gut microbial composition prior to the intervention (Table 4 and Figure S1: Alpha and beta diversity baseline).

#### 3.1.2. Taxonomic Composition Profiling

The overall gut microbiota composition at the phylum and genus levels was broadly similar between the two groups at baseline, dominated by *Bacteroidetes* and *Firmicutes*. The subsequent LEfSe analysis was presented as an exploratory, hypothesis-generating assessment of group-specific taxonomic signatures at baseline rather than primary evidence of between-group differences (See Figure S2 for taxonomic composition profiles).

#### 3.1.3. LEfSe Analysis of Differentiated Species of Intestinal Flora

**Figure 3:**
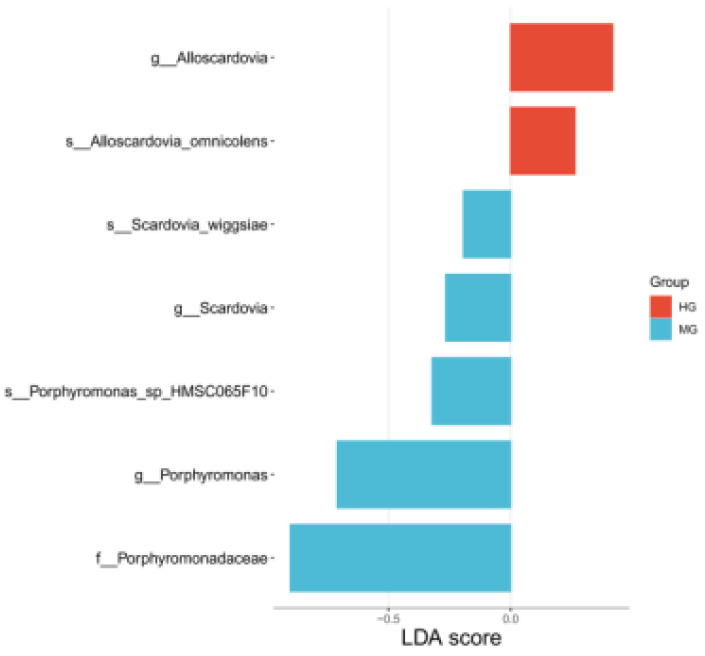
Plot of LDA scores for differentiated species (baseline).

LEfSe analysis (LDA > 2.0, Figure 3) identified significantly enriched microbial taxa in each group: HG: *Alloscardovia* (genus) and *Alloscardovia_omnicolens* (species); MG: *Scardovia* (genus), *Scardovia_wiggsiae* (species), *Porphyromonas* (genus), *Porphyromonas_sp_ HMSC065F10* (species), and *Porphyromonadaceae* (family). The corresponding comparison-specific, taxonomic-rank-stratified FDR-corrected results are provided in Table S4: Comparison-specific, taxonomic-rank-stratified Benjamini–Hochberg FDR correction for reported LEfSe-derived taxonomic findings.

#### 3.1.4. Differential Genes and Metabolic Pathways in Intestinal Flora

**Figure 4:**
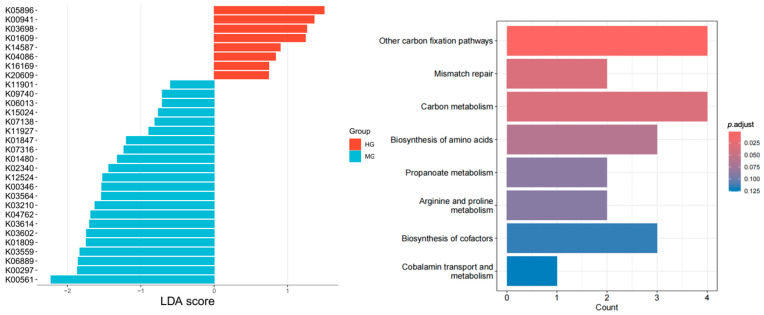
Differential genes and metabolic pathways (baseline).

Differential gene analysis (Figure 4) identified 22 significantly different genes between HG and MG via LEfSe. KEGG pathway enrichment analysis of these genes revealed significant enrichment in eight metabolic pathways: other carbon fixation pathways, mismatch repair, carbon metabolism, biosynthesis of amino acids, propanoate metabolism, arginine and proline metabolism, biosynthesis of cofactors, and cobalamin transport and metabolism.

### 3.2. Differences in Intestinal Flora in the HG from Baseline to Post-Intervention

#### 3.2.1. Alpha and Beta Diversity Analysis

In the healthy group (HG), the 12-week exercise intervention resulted in a significant decrease in the Shannon index of alpha diversity (*p* < 0.05), while no significant shift was observed in the overall microbial structure (beta diversity) (Table 4 and Figure S3: HG—alpha and beta diversity (baseline vs. post-intervention)).

#### 3.2.2. Taxonomic Composition Profiling

Figure S4 depicts the taxonomic composition in the HG at baseline vs. post-intervention. Post-intervention, specific alterations were observed in the taxonomic composition of the HG gut microbiota. At the phylum level, a marked increase in the relative abundance of *Proteobacteria* was evident. This shift was clearly defined at finer taxonomic resolutions: the genus Escherichia exhibited a substantial increase, which was correspondingly reflected at the species level by a significant rise in *Escherichia_coli*. These distinct compositional shifts provided clear targets for the subsequent identification of key biomarkers.

#### 3.2.3. LEfSe Analysis of Differentiated Species of Intestinal Flora

**Figure 5:**
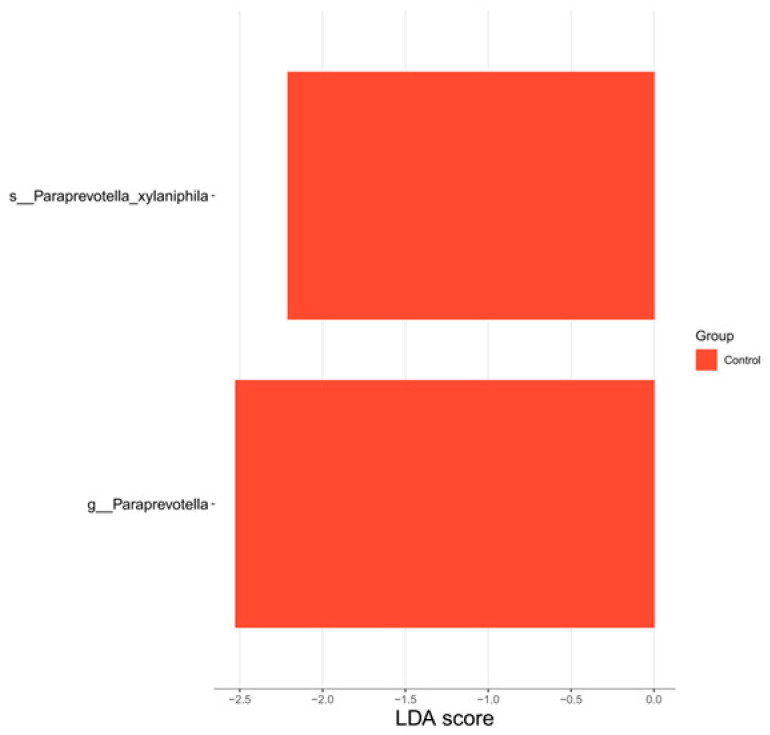
HG—plot of LDA scores for differentiated species (baseline vs. post-intervention).

LEfSe analysis (Figure 5) identified two microbial taxa significantly enriched in the HG post-intervention: *Paraprevotella* (genus) and *Paraprevotella_xylaniphila* (species).

#### 3.2.4. Differential Genes and Metabolic Pathways in Intestinal Flora

**Figure 6:**
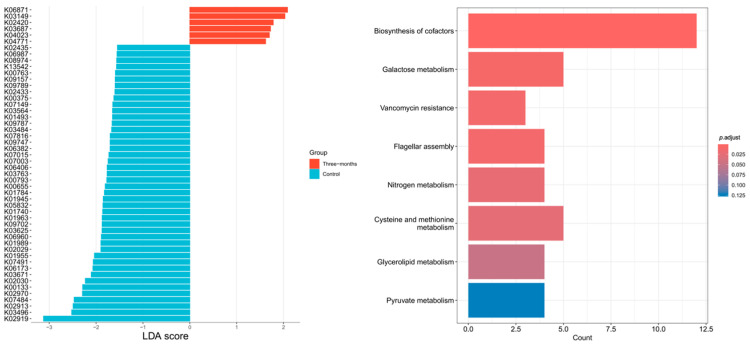
HG—differential genes and metabolic pathways (baseline vs. post-intervention).

Differential gene analysis in the HG (Figure 6) identified 50 significantly different genes between baseline and post-intervention. KEGG pathway enrichment analysis revealed significant enrichment in eight pathways: biosynthesis of cofactors, galactose metabolism, vancomycin resistance, flagellar assembly, nitrogen metabolism, cysteine and methionine metabolism, glycerolipid metabolism, and pyruvate metabolism.

### 3.3. Differences in Intestinal Flora in the MG from Baseline to Post-Intervention

#### 3.3.1. Alpha and Beta Diversity Analysis

In the MG, the 12-week exercise intervention did not induce significant changes in either the alpha diversity or the overall structure (beta diversity) of the gut microbiota (all *p* > 0.05) (Table 4 and Figure S5: MG—alpha and beta diversity (baseline vs. post-intervention)).

#### 3.3.2. Taxonomic Composition Profiling

Post-intervention, compositional alterations were observed in the taxonomic composition of the MG gut microbiota. At the phylum level, the relative abundance of Firmicutes, known for their role in energy harvesting, increased, with a corresponding decrease in *Bacteroidetes*. This structural reorganization was reflected at the species level by a substantial rise in the anti-inflammatory bacterium *Faecalibacterium prausnitzii*, while core species, including *Bacteroides uniformis* and *Escherichia coli*, remained stable (Figure S6: MG—taxonomic composition profiling (base-line vs. post-intervention)). These compositional shifts provided a basis for subsequent functional analysis.

#### 3.3.3. LEfSe Analysis of Differentiated Species of Intestinal Flora

**Figure 7:**
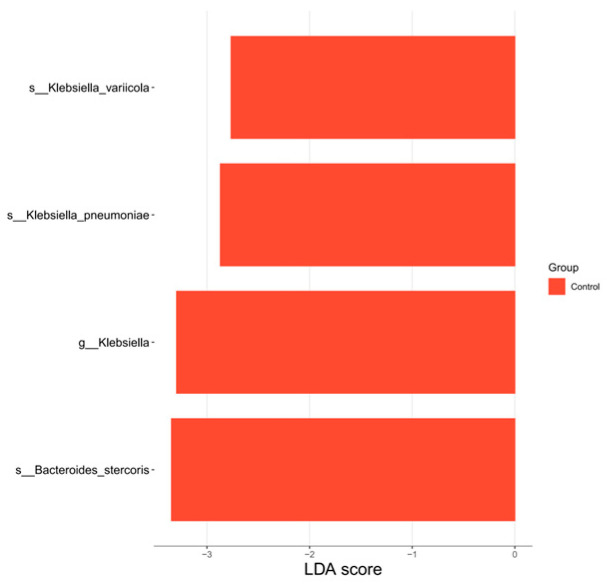
MG—plot of LDA scores for differentiated species (baseline vs. post-intervention).

LEfSe analysis (Figure 7) identified four microbial taxa significantly enriched in the MG post-intervention: *Klebsiella_variicola* (species), *Klebsiella_pneumoniae* (species), *Klebsiella* (genus), and *Bacteroides_stercoris* (species). These enriched taxa should be interpreted cautiously. In particular, the enrichment of *Klebsiella pneumoniae* and *Klebsiella variicola* in the MG may represent an unfavorable component of the observed taxonomic shifts.

#### 3.3.4. Differential Genes and Metabolic Pathways in Intestinal Flora

**Figure 8:**
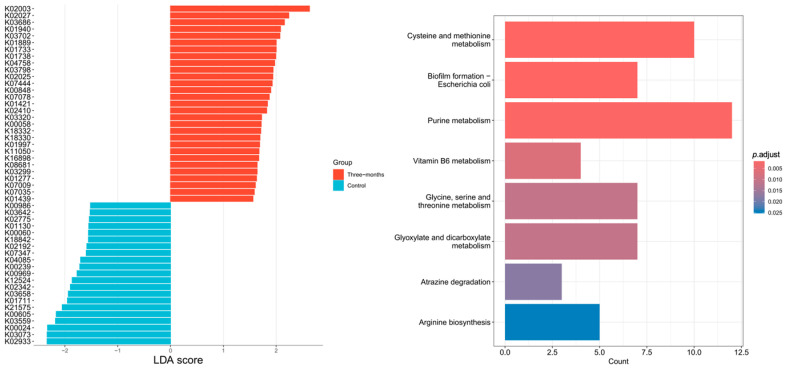
MG—differential genes and metabolic pathways (baseline vs. post-intervention).

Figure 8 presents the LDA scores of differential genes in the MG at baseline and post-intervention. A total of 50 differential genes were identified, with 29 enriched post-intervention and 21 at baseline. KEGG pathway enrichment analysis revealed eight significantly enriched metabolic pathways, including cysteine and methionine metabolism, purine metabolism, vitamin B6 metabolism, glycine, serine and threonine metabolism, arginine biosynthesis, biofilm formation (*Escherichia coli*), glyoxylate and dicarboxylate metabolism, and atrazine degradation. These findings suggest that exercise may modulate amino acid and vitamin metabolism, as well as microbial biofilm-related pathways.

### 3.4. Differences in Intestinal Flora Between the Two Groups at Post-Intervention

#### 3.4.1. Alpha and Beta Diversity Analysis

**Figure 9:**
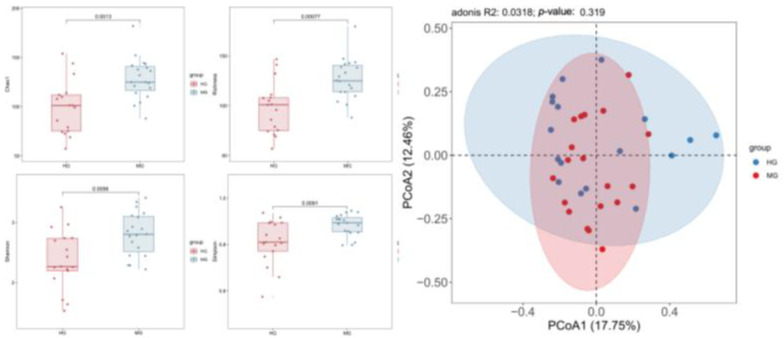
Alpha and beta diversity (post-intervention).

Post-intervention comparison revealed a significant divergence in gut microbiota diversity between the groups (*p* < 0.05). The malnourished group (MG) demonstrated significantly higher microbial richness (Chao1 index) and overall diversity (Shannon index) than the healthy group (HG), whereas the HG showed significantly greater community evenness (Simpson index) (Table 4 and Figure 9).

#### 3.4.2. Taxonomic Composition Profiling

Post-intervention, differences in taxonomic composition were observed between the HG and MG. Although the core phylum remained generally similar, *Faecalibacterium prausnitzii*, a bacterial species previously associated with beneficial gut ecological characteristics, showed relatively higher abundance in the MG compared with the HG. In contrast, the HG remained characterized by relatively higher abundances of *Bacteroides vulgatus* and *Escherichia coli* (Figure S7: Taxonomic composition profiling (post-intervention)). These observations may reflect differences in microbiota profiles according to nutritional status during the study period. These findings should be interpreted cautiously, given the absence of a non-intervention control group.

#### 3.4.3. LEfSe Analysis of Differentiated Species of Intestinal Flora

**Figure 10:**
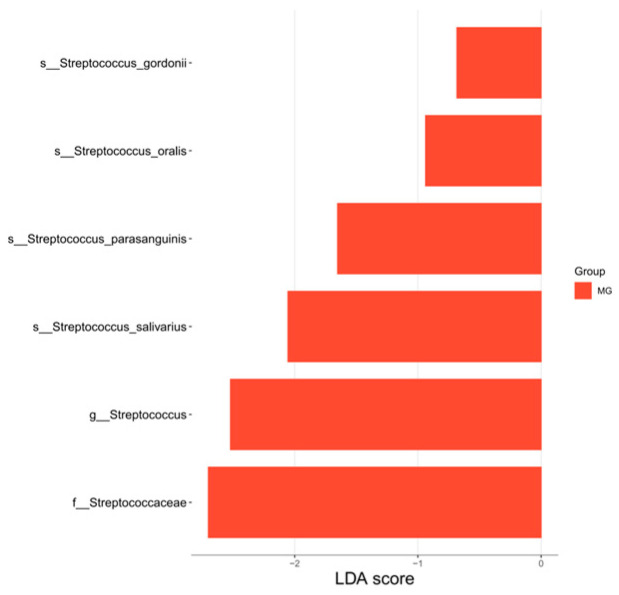
Plot of LDA scores for differentiated species (post-intervention).

Figure 10 shows the LDA score plot identifying significantly enriched taxa in the MG compared to the HG after 12 weeks of exercise intervention. Six significantly enriched features belonging to the *Streptococcaceae* family were identified: the family *Streptococcaceae* itself, the genus *Streptococcus*, and the species *Streptococcus_salivarius*, *Streptococcus_parasanguinis*, and *Streptococcus_oralis*. This enrichment suggests a potential association between these *Streptococcaceae*-related taxa and the gut environment of the MG following the exercise intervention.

#### 3.4.4. Differential Genes and Metabolic Pathways in Intestinal Flora

**Figure 11:**
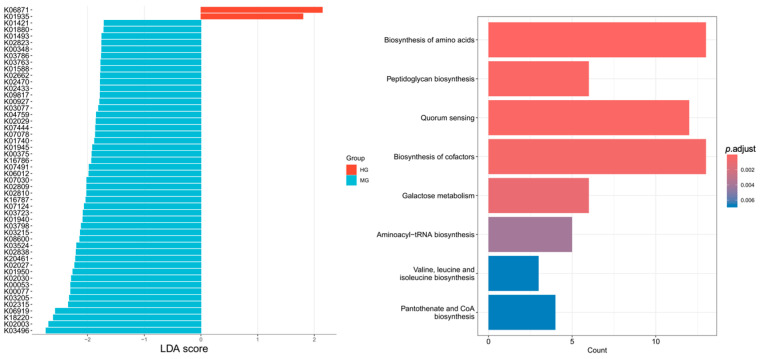
Differential genes and metabolic pathways (post-intervention).

Figure 11 presents the analysis of differential genes in the gut microbiota between the HG and MG following the 12-week exercise intervention. A total of 50 differential genes were identified, with 2 enriched in the HG and 48 enriched in the MG. KEGG pathway enrichment analysis of these 50 genes revealed significant enrichment in 8 metabolic pathways: biosynthesis of amino acids; peptidoglycan biosynthesis; quorum sensing; biosynthesis of cofactors; galactose metabolism; aminoacyl-tRNA biosynthesis; valine, leucine, and isoleucine biosynthesis; and pantothenate and CoA biosynthesis.

### 3.5. Safety

No adverse events or serious health complications were reported during the 12-week intervention period across both groups.

## 4. Discussion

The purpose of this study was to investigate the differences in gut flora between malnourished and healthy older adults and the effects of a 12-week exercise intervention on both groups. The results showed significant differences in gut flora species between malnourished and healthy older adults. After the 12-week study period, microbiota differences were observed in the malnourished group, although these changes should be interpreted cautiously given the study design.

### 4.1. Differences in Intestinal Flora Between HG and MG Before Intervention

The absence of significant alpha and beta diversity differences between groups at baseline likely stems from two key factors: (1) age-related microbiota stabilization in elderly populations, where diminished immune and metabolic flexibility constrains community restructuring [19], and (2) malnutrition’s primary impact via non-microbiota-dependent pathways (e.g., systemic metabolic dysregulation) [20]. Crucially, the host’s compensatory physiological adaptations to nutrient deficits—such as enhanced nutrient recycling—may further buffer against diversity shifts, underscoring gut ecosystem resilience even under nutritional stress.

LEfSe analysis revealed stark functional contrasts: *Alloscardovia* enrichment in the HG implies preserved metabolic homeostasis, potentially through modulating host glycometabolism [21,22]. In the MG, the prominence of *Faecalibacterium* as a dominant SCFA producer (butyrate > acetate > propionate) may indicate potential host-relevant functions. SCFAs have been reported to support gut barrier function, modulate inflammatory signaling, and serve as an energy source for colonocytes, which may be relevant when considering microbiota changes in malnutrition [23,24,25,26]. Concurrent *Porphyromonas* enrichment indicates protein salvage adaptation, where proteolytic activity liberates amino acids from endogenous proteins (e.g., mucins) to sustain energy production during nutrient scarcity [27]. However, because SCFAs, epithelial barrier markers, and inflammatory indicators were not directly measured in the present study, any implications for gut barrier function, inflammatory signaling, or host protection should be regarded as hypothetical rather than confirmed.

Pathway alterations (carbon metabolism and amino acid biosynthesis) may reflect coordinated microbiota–host coadaptation. Carbon flux redirection accelerates energy harvest from limited carbohydrates, prioritizing ATP generation over anabolism [28,29], while essential amino acid synthesis (valine/leucine/isoleucine) compensates for dietary deficits, providing substrates for gluconeogenesis and muscle preservation [28,29]. Synergistically, these pathways stabilize microbial communities by optimizing nutrient digestion and modulating nitrogen cycling—elevating ammonium assimilation for microbial biomass production [21].

This metabolic pattern may be consistent with ecological adaptations described in nutrient-poor environments and may suggest a potential microbiota-related response to malnutrition [30] through energy rescue (SCFAs and carbon metabolism), protein sparing (amino acid biosynthesis), and barrier–immune crosstalk (SCFA-mediated immunomodulation).

### 4.2. Differences in Intestinal Flora in the HG Before and After Exercise Intervention

Exercise intervention significantly reduced gut microbiota diversity (Shannon index) in the HG, likely driven by exercise-induced gut microenvironment alterations (e.g., pH and redox potential shifts) that selectively inhibit specific taxa [31]. Although beta diversity exhibited non-significant structural changes, this may reflect either the 12-week intervention’s insufficient duration for core microbiota restructuring (e.g., Bacteroidetes stability) or the resilience of dominant phyla like Firmicutes buffering ecosystem disturbance.

Post-intervention species composition shifted markedly: *Proteobacteria* abundance surged from 6.0% to 15.2%, potentially linked to exercise-modulated redox dynamics [32]. Notably, *Escherichia* (pathogen-associated) and *Eubacterium* (SCFA-producing) increased from low baselines to 12.9% and 7.1%, respectively, suggesting concurrent microbial adaptation to gut environmental changes, with possible implications for SCFA-related metabolism [33]. LEfSe analysis identified enrichment of *Paraprevotella* and *P. xylaniphila* (Bacteroidetes), which synergistically degrade complex carbohydrates (e.g., xylan) and have been reported to generate acetate/propionate/butyrate and may be relevant to gut barrier integrity and systemic metabolism [34]. Our findings in the HG differ from those of Morita et al. [14], who reported an increase in intestinal *Bacteroides* after a 12-week brisk-walking-based aerobic exercise program in healthy elderly women. A similar pattern was not clearly observed in our HG. This difference may be related to differences in participant characteristics, nutritional status stratification, and microbiome assessment methods between the two studies.

KEGG pathway analysis revealed three key metabolic adaptations: cofactor biosynthesis upregulation promotes vitamin/coenzyme synthesis [35], enhancing energy metabolism and antioxidant defenses; galactose metabolism activation improves lactose-derived energy harvest [36]; and pyruvate metabolism amplification optimizes glycolytic flux and energy yield [37]. Crucially, these pathways operate synergistically: cofactors elevate enzymatic efficiency for galactose/pyruvate processing, while galactose-derived substrates fuel pyruvate-driven ATP synthesis. This metabolic pattern may suggest that exercise-associated microbiota modulation is linked to changes in microbial metabolic potential.

### 4.3. Differences in Intestinal Flora in the MG Before and After Exercise Intervention

Exercise intervention did not significantly alter alpha and beta diversity in the MG, indicating limited structural impact on microbiota communities. This resilience likely stems from malnutrition-adapted microbiota stability, where core communities buffer environmental perturbations. The 12-week intervention may have been insufficient in intensity or duration to overcome this buffering capacity.

Species composition shifts revealed functional adaptations: *Bacteroidetes* decreased (57.6% → 48.2%), suggesting attenuated carbohydrate metabolism capacity [34]; *Firmicutes* increased (28.6% → 38.8%), indicating enhanced energy harvest efficiency [38]; concurrent rises in *Eubacterium* (to 6.0%) and *Faecalibacterium_prausnitzii* (7.9% → 12.6%) may reflect shifts in SCFA-related taxa, broadly consistent with the pattern observed in the HG.

LEfSe analysis identified a mixed pattern of taxonomic enrichment in the MG. While the increased abundance of *Bacteroides stercoris* may be functionally relevant to SCFA production [15,39], and may have functional relevance in relation to *Faecalibacterium*, as suggested in previous studies [40], the enrichment of *Klebsiella pneumoniae* and *Klebsiella variicola* should be interpreted with caution. In a malnourished population aged 80 years and above, the increase in these Klebsiella-related taxa should not be interpreted as a favorable change. Members of the *Klebsiella* genus can occur as asymptomatic gastrointestinal colonizers, and changes in host immune status may influence their abundance [41,42,43]. However, *K. pneumoniae* is widely regarded as an opportunistic pathogen, and *K. variicola* has also emerged as a clinically relevant human pathogen [42,44]. Accordingly, these post-intervention taxonomic shifts may reflect not only potentially adaptive restructuring of the gut microbiota but also a potentially unfavorable ecological signal. The present study did not assess infection-related or immune-related clinical markers, and therefore, the clinical significance of this finding cannot be determined from the current data alone.

KEGG pathway alterations suggested potential differences in predicted microbial metabolic functions. Pathways related to amino acid metabolism (methionine/arginine) [45,46]. SCFA-associated metabolism and biofilm regulation may be relevant to antioxidant processes, epithelial maintenance, and microbial ecological interactions, according to previous literature [47,48]. However, because microbial metabolites, inflammatory biomarkers, and gut barrier-related markers were not directly measured in this study, these functional interpretations should be regarded as hypothetical and exploratory rather than confirmed biological effects.

### 4.4. Differences in Intestinal Flora Between HG and MG After Intervention

Our findings suggest that post-intervention gut microbiota profiles differed between the MG and HG after the 12-week study period. In the MG, higher post-intervention microbial richness, diversity, and predicted metabolic functional capacity were observed after the 12-week study period. However, because the study lacked a non-exercise control group, these observations should not be interpreted as definitive effects attributable to exercise alone. These observations may indicate that aerobic exercise is associated with microbiota patterns that differ according to nutritional status in very old adults. However, whether exercise directly contributes to these differences remains uncertain, potentially by favoring adaptable taxa capable of thriving in nutrient-limited conditions [49]. Conversely, the HG microbiota maintained higher uniformity post-intervention, characterized by a stable distribution of dominant taxa like *Bacteroidetes*, indicative of preserved metabolic homeostasis. In the MG, however, the enrichment of *Streptococcus* appeared to disrupt this uniformity, potentially establishing a functionally dominant niche.

Post-intervention compositional differences with potential relevance to gut health were observed. The MG showed an increase in *Firmicutes* (involved in SCFA production, critical for intestinal barrier integrity and immune regulation), alongside a decrease in *Bacteroidetes* [38]. While *Bacteroidetes* abundance remained higher in the HG, consistent with their established role in gut metabolism, anti-inflammation, and immune modulation within a healthy environment, the significant enrichment of *Faecalibacterium_prausnitzii* in the MG is particularly noteworthy [40]. This bacterium is renowned for its anti-inflammatory properties and enhancement of barrier function via SCFA production and immune modulation [26,39]. While the enrichment of *Faecalibacterium prausnitzii* in the MG may be of biological interest, these findings should be interpreted cautiously and not as definitive evidence that exercise ameliorated gut dysbiosis or inflammation.

LEfSe analysis further highlighted enrichment of *Streptococcaceae-* and *Streptococcus-related taxa* in the MG post-exercise. This finding should be interpreted cautiously. In malnourished hosts, changes in nutritional or luminal substrate conditions may influence colonic microbial ecology, and the biological meaning of *streptococcal* enrichment is therefore likely to be context-dependent rather than uniformly favorable [50]. Although some species, such as *Streptococcus salivarius*, have been described as commensals with ecological roles in mucosal environments, the genus *Streptococcus* also includes members with clear pathogenic potential, exemplified by *Streptococcus pneumoniae* [51,52]. Therefore, the genus-level enrichment observed in the present study should not be interpreted as inherently beneficial or as direct evidence of improved immune homeostasis or anti-inflammatory effects. Rather, the identified Streptococcus-related signals are better regarded as descriptive taxonomic differences associated with the post-intervention microbiota profile, while biological interpretation should remain limited to the species or functional level.

From a functional perspective, KEGG pathway analysis suggested differences in predicted microbial pathways in the MG, particularly amino acid biosynthesis (including BCAAs) and quorum sensing. Altered amino acid biosynthesis may be relevant to host immune function and metabolic balance, a critical benefit in malnutrition [48,53]. Increased BCAAs (valine, leucine, and isoleucine) point to exercise promoting cellular repair, protein synthesis, and energy balance restoration [54]. Furthermore, enrichment in quorum sensing and cofactor synthesis suggests exercise fosters microbial cooperation, enhancing collective metabolic function, host immune defenses, and overall gut ecosystem health [35]. However, because microbial metabolites such as SCFAs and host inflammatory or barrier-related biomarkers were not directly measured in this study, the implications of these predicted functional shifts for immune function, epithelial repair, or gut barrier integrity should be regarded as hypothetical rather than confirmed effects.

Taken together, the present study extends current research on the gut–exercise–nutrition axis in very old adults by focusing on individuals aged 80 years and above and by examining microbiota differences according to nutritional status over a 12-week aerobic exercise intervention. The longitudinal design, combined with gut microbiota profiling and functional pathway analysis, provides a more integrated view of how exercise may be associated with microbial diversity, composition, and predicted metabolic function in malnourished older adults. These features strengthen the contribution of the study to the emerging literature on microbiota-oriented, non-pharmacological strategies in geriatric care.

## 5. Conclusions

In conclusion, this assessor-blinded study stratified by nutritional status observed gut microbiota differences over a 12-week period in older adults aged 80 years and above who participated in an aerobic exercise program. While baseline microbial diversity showed minimal differences between the healthy and malnourished groups, the post-intervention assessment showed that the malnourished group had higher microbial richness and diversity than the healthy group, along with distinct taxonomic and predicted functional profiles. Functional gene enrichment also suggested differences in pathways related to amino acid biosynthesis, protein synthesis, and quorum sensing, indicating possible differences in microbial functional potential. These findings should be interpreted as descriptive associations observed during the study period rather than definitive effects of the exercise program. Because no non-intervention control group was included, the observed microbiota differences cannot be conclusively attributed to the exercise intervention itself. Direct effects on immune modulation and gut barrier integrity were not measured in this study.

In contrast, the healthy group showed more limited microbiota differences over the same 12-week period. Within the constraints of this study design, the microbiota differences observed after 12 weeks appeared more evident in the malnourished group; however, causality remains uncertain because the study lacked a non-exercise control group, and natural temporal variation, dietary changes, and facility-related environmental influences could not be excluded.

### 5.1. Practical Recommendations

Based on the findings of this study, moderate-intensity aerobic exercise, specifically brisk walking at 40–60% heart rate reserve (HRR), performed three times per week for 12 weeks, may be considered a feasible non-pharmacological strategy warranting further evaluation in malnourished adults aged 80 years and above, rather than a confirmed approach to improve gut microbiota composition and metabolic function. Structured walking programs may represent a feasible and low-risk intervention model for future investigation in older adults with nutritional vulnerability. However, the present findings should not be interpreted as evidence of confirmed clinical efficacy for improving gut dysbiosis or related health outcomes.

To ensure safety and maximize effectiveness, exercise programs should be tailored to individual functional capacity, supervised by trained personnel, and supported by real-time monitoring tools such as heart rate watches. Additionally, nutritional screening tools such as the MNA-SF may help identify subgroups for future exercise-based microbiota research. Community-based interventions that integrate exercise, nutrition, and microbiota education may further enhance adherence and outcomes. The present findings may contribute to the growing body of exploratory research examining the relationship between physical activity, nutritional status, and gut microbiota profiles in very old adults. Further controlled studies are required before specific microbiota-related or clinical recommendations can be established.

### 5.2. Limitations

This study has several limitations that should be acknowledged.

First, a major limitation of this study is the substantial attrition from enrollment to final analysis, with only 36 of 129 participants completing the 12-week intervention and contributing to the final analysis. Although no statistically detectable differences were observed between completers and non-completers or between study groups in the available attrition analyses, the overall attrition rate remained high at 72.1%. True differences may have gone undetected because of limited statistical power, and substantial attrition bias cannot be excluded. This level of attrition may have undermined both the internal and external validity of the study, and the primary microbiome analyses, which were based on a per-protocol sample, should therefore be interpreted with caution. Sensitivity analyses of available continuous clinical and nutritional outcomes suggested that the overall pattern of non-sequencing findings was not materially altered under a conservative missing-data assumption; however, comparable analyses were not feasible for sequencing-based microbiome endpoints because many dropouts lacked post-intervention stool samples.

Second, the study was conducted in a single community-based elderly care facility in northern China, potentially introducing selection bias and restricting the geographic and ethnic diversity of the sample population.

Third, several important confounding factors in gut microbiota research were not systematically assessed or controlled, including dietary intake, antibiotic or probiotic use, comorbidities, and concomitant medications. In this elderly population, strict control of daily diet and clinically indicated medications was not feasible in practice and, in some cases, would not have been ethically appropriate. Participants were therefore asked to maintain their usual lifestyle and treatment routines during the intervention. Nevertheless, the lack of detailed assessment or adjustment for these factors limits the interpretation of the microbiota findings and means that residual confounding cannot be excluded.

Fourth, although this study employed high-throughput sequencing and functional pathway prediction, the lack of direct measurements of microbial metabolites (e.g., SCFAs) or inflammatory biomarkers limits definitive conclusions about host–microbiota interactions. Furthermore, despite applying hierarchical FDR correction to LEfSe outputs, the differential taxonomic and predicted functional analyses remain exploratory due to the modest final sample size and the number of comparisons performed. These findings should, therefore, be interpreted as supportive, hypothesis-generating signals rather than primary, confirmatory evidence.

Finally, the relatively short duration of the intervention (12 weeks) may not fully capture the long-term effects of aerobic exercise on gut microbiota and overall health outcomes.

Future studies with larger, more diverse populations, longer follow-up periods, and integrated multi-omics approaches are warranted to validate and expand upon these findings.

## Figures and Tables

**Table 1:** Participants’ characteristics.

			MG (*n* = 69)	HG (*n* = 60)
Descriptive Characteristics	Sex	Male	24 (34.8%)	28 (46.7%)
		Female	45 (65.2%)	32 (53.3%)
	Age (years)		85.2 ± 3.1	85.7 ± 3.1
	BMI (kg·m^−2^)		23.7 ± 3.3	24.4 ± 3.3

**Table 2:** Mini Nutritional Assessment Short-Form (MNA-SF).

Question	Assessment Criteria and Score	
Weight loss during the last 3 months?	0 = >3 kg	(0)
	1 = Does not know	(1)
	2 = 1~3 kg	(2)
	3 = No weight loss	(3)
BMI (kg/m^2^)	0 = <19	(0)
	1 = 19~21	(1)
	2 = 21~23	(2)
	3 = >23	(3)
Has suffered psychological stress or acute disease in the past 3 months?	0 = NO	(0)
	2 = YES	(2)
Mobility?	0 = Bed or chair-bound	(0)
	1 = Able to get out but not go out	(1)
	2 = Goes out	(2)
Neuropsychological problems?	0 = Severe dementia/depression	(0)
	1 = Mild dementia	(1)
	2 = No psychological problems	(2)
Has food intake declined over the past 3 months?	0 = Severe decrease in food intake	(0)
	1 = Moderate decrease in food intake	(1)
	2 = No decrease in food intake	(2)

**Table 3:** Participant characteristics (post-intervention).

			MG (*n* = 19)	HG (*n* = 17)	*p*
Descriptive Characteristics	Sex	Male	10 (52.6%)	10 (58.8%)	0.75
		Female	9 (47.4%)	7 (41.2%)	
	Age (years)		83.4 ± 3.0	83.6 ± 2.9	0.83
	BMI (kg·m^−2^)		22.7 ± 2.6	24.1 ± 2.5	0.083

**Table 4:** Summary of primary diversity findings.

Comparison	Alpha Diversity	Beta Diversity	Main Finding
Baseline HG vs. MG	NS ^1^	NS	Similar overall diversity at baseline
HG: baseline to post-intervention	Shannon index decreased; others NS	NS	Limited within-group change
MG: baseline to post-intervention	NS	NS	No detectable within-group change
Post-intervention HG vs. MG	MG has higher richness/diversity; HG has higher evenness	Significant	Clearest diversity difference after 12 weeks

^1^ Abbreviations: NS, not significant. HG, healthy group. MG, malnourished group.

## Data Availability

The data used in the article have been uploaded to the Supplementary Materials; further inquiries can be directed to the corresponding author.

## Supplementary Materials

The following supporting information can be downloaded at https://www.mdpi.com/article/10.3390/nu18101627/s1, Figure S1: Alpha and beta diversity (baseline); Figure S2: Taxonomic composition profiling (baseline); Figure S3: HG—alpha and beta diversity (baseline vs. post-intervention); Figure S4: HG—taxonomic composition profiling (pre- vs. post-intervention); Figure S5: MG—alpha and beta diversity (baseline vs. post-intervention); Figure S6: MG—taxonomic composition profiling (baseline vs. post-intervention); Figure S7: Taxonomic composition profiling (post-intervention); Table S1: Attrition analysis; Table S2: Sensitivity analysis using baseline observation carried forward (BOCF) for available continuous clinical/nutritional outcomes; Table S3: Baseline objective body-composition indicators according to nutritional status grouping and their correlations with baseline MNA-SF scores; Table S4: Comparison-specific, taxonomic-rank-stratified Benjamini–Hochberg FDR correction for reported LEfSe-derived taxonomic findings.
