# Supplementary material for: Gut Microbiota Changes Following Aerobic Exercise in Malnourished Octogenarians: An Assessor-Blinded Intervention Study Stratified by Nutritional Status
Source: Nutrients. 2026 May 20;18(10):1627. doi: 10.3390/nu18101627 (PMC13209846; doi:10.3390/nu18101627)
Supplement: Supplementary file 1 [file nutrients-18-01627-s001.zip › Supplementary Materials.pdf]

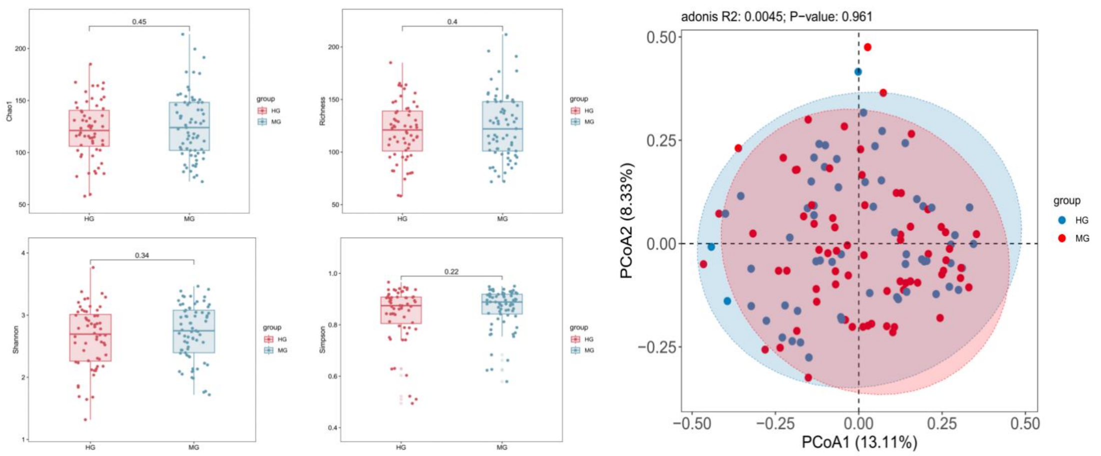

Alpha Diversity **Beta Diversity**

Figure S 1. Alpha and Beta Diversity (baseline)

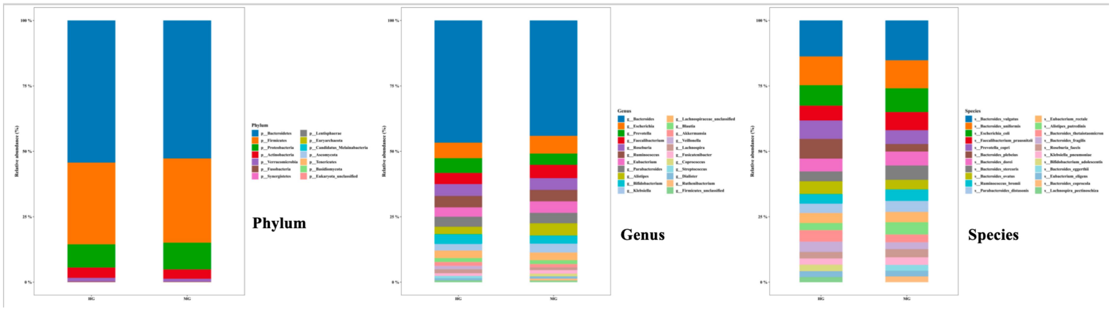

Figure S 2. Taxonomic Composition Profiling (baseline)

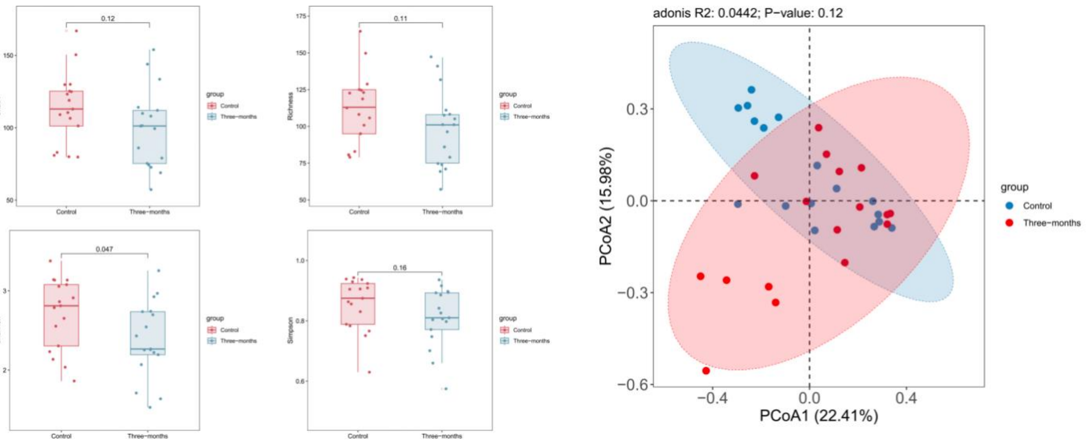

Alpha Diversity **Beta Diversity**

Figure S 3. HG-Alpha and Beta Diversity (baseline vs. post-intervention)

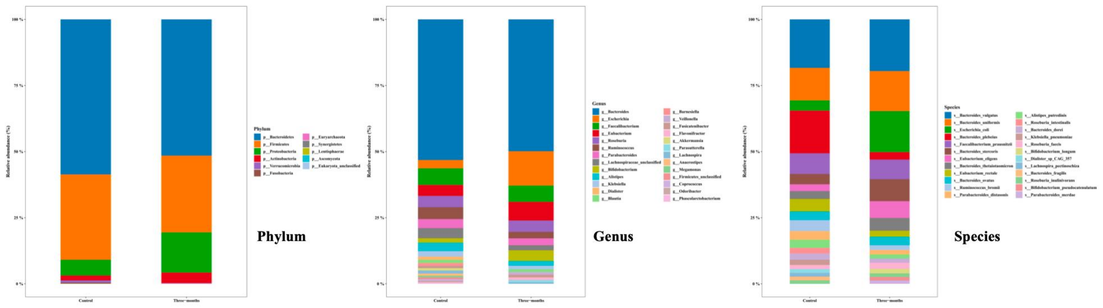

Figure S 4. HG-Taxonomic Composition Profiling (baseline vs. post-intervention)

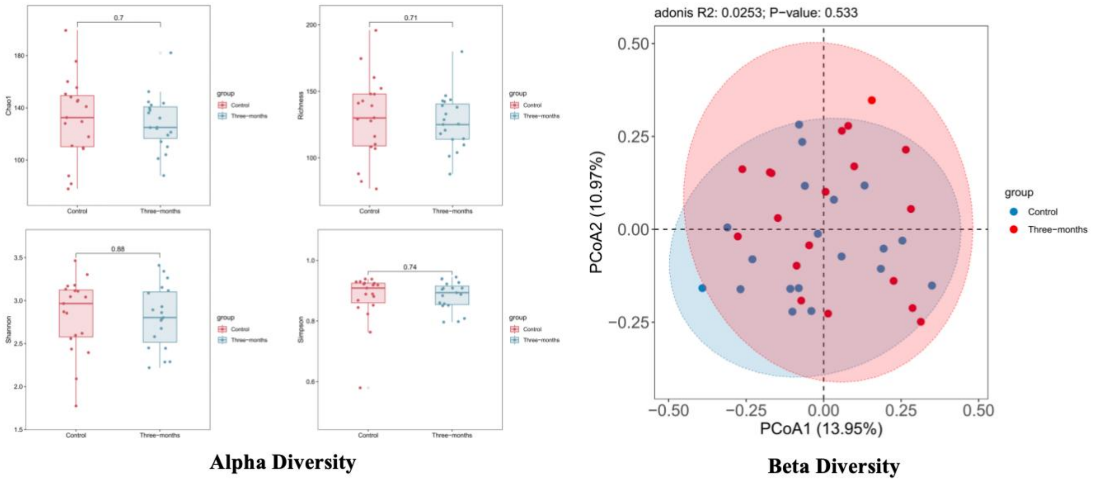

Figure S 5. MG-Alpha and Beta Diversity (baseline vs. post-intervention)

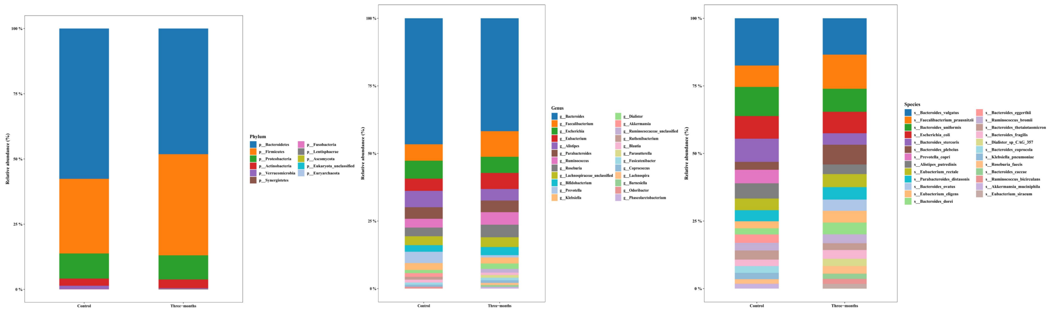

Figure S 6. MG-Taxonomic Composition Profiling (baseline vs. post-intervention)

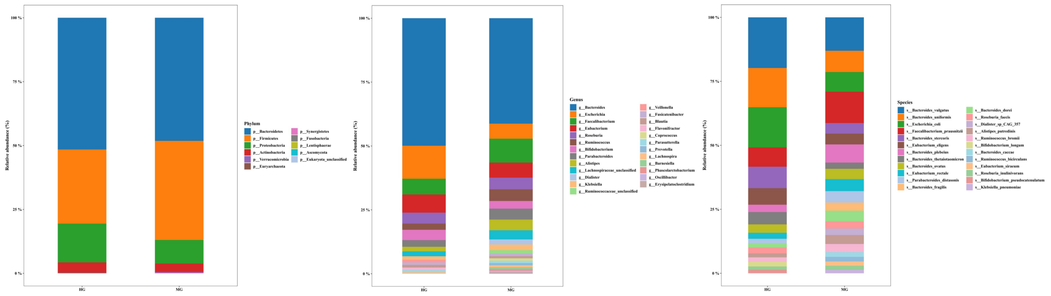

Figure S 7. Taxonomic Composition Profiling (post-intervention

Table S1. Attrition analysis

Section /Variable	Completers (n = 36)	Non-completers (n = 93)	HG	MG	P value
**Baseline characteristics of completers and non-completers**					
MG, n (%)	19 (52.8%)	50 (53.8%)			1.000
Female, n (%)	20 (55.6%)	57 (61.3%)			0.556
Age (years), M ± SD	85.47 ± 3.05	85.40 ± 3.11			0.902
BMI (kg/m²), M ± SD	23.24 ± 2.85	24.32 ± 3.42			0.075
**Completion and dropout stage by study group**					
Completed, n (%)			17 (28.3%)	19 (27.5%)	1.000
Attrition, n (%)			43 (71.7%)	50 (72.5%)	1.000
Baseline dropout, n (%)			24 (40.0%)	30 (43.5%)	0.916
Post-intervention dropout, n (%)			19 (31.7%)	20 (29.0%)	0.916
**Overall reasons for non-completion by study group (non-completers only)**					
Time conflicts, n (%)			9 (20.9%)	12 (24.0%)	0.956
Personal circumstances, n(%)			7 (16.3%)	8 (16.0%)	0.956
Attendance < 75%, n (%)			9 (20.9%)	9 (18.0%)	0.956
Disease, n (%)			7 (16.3%)	6 (12.0%)	0.956
Other reasons, n (%)			11 (25.6%)	15 (30.0%)	0.956
**Reasons for dropout before the intervention**					
Time conflicts, n (%)			9 (37.5%)	12 (40.0%)	0.975
Personal circumstances, n(%)			7 (29.2%)	8 (26.7%)	0.975
Other reasons, n (%)			8 (33.3%)	10 (33.3%)	0.975
**Reasons for non-completion after the intervention**					
Attendance < 75%, n (%)			9 (47.4%)	9 (45.0%)	0.759
Disease, n (%)			7 (36.8%)	6 (30.0%)	0.759
Other reasons, n (%)			3 (15.8%)	5 (25.0%)	0.759

For subsections with more than two categories, the reported p value represents the overall between-group comparison for that subsection rather than the comparison of an individual row.

Table S2. Sensitivity analysis using baseline observation carried forward (BOCF) for available continuous clinical/nutritional outcomes.

Outcome/ Analysis	Group	n	Baseline (M ± SD)	Post (M ± SD)	Change (M ± SD)	P value
			**BMI (kg/m²)**			
**Per-protocol**	HG	17	23.92 ± 1.90	24.05 ± 2.12	0.13 ± 0.80	0.517
	MG	19	22.64 ± 3.43	22.69 ± 2.77	0.05 ± 1.31	0.863
	HG&MG	HG=17 MG=19	HG 0.13 vs MG 0.05			0.832
**Sensitivity analysis (BOCF)**	HG	60	24.41 ± 2.40	24.41 ± 2.50	-0.00 ± 0.76	0.987
	MG	69	23.67 ± 3.90	24.02 ± 3.73	0.34 ± 2.26	0.211
	HG&MG	HG=60 MG=69	HG -0.00 vs MG 0.34			0.237
			**Weight (kg)**			
**Per-protocol**	HG	16	61.09 ± 9.42	61.43 ± 9.37	0.34 ± 2.41	0.583
	MG	17	59.74±13.01	59.84 ±11.28	0.10 ± 5.13	0.937
	HG&MG	HG=16 MG=17	HG 0.34 vs MG 0.10			0.865
**Sensitivity analysis (BOCF)**	HG	50	64.18 ± 9.88	64.34 ± 9.84	0.16 ± 1.39	0.415
	MG	36	58.90±13.96	59.88 ± 12.70	0.98 ± 5.68	0.306
	HG&MG	HG=50 MG=36	HG 0.16 vs MG 0.98			0.401
			**MNA-SF score**			
**Per-protocol**	HG	17	11.88 ± 0.93	11.18 ± 0.81	-0.71± 1.05	0.013
	MG	19	9.21 ± 1.23	10.53 ± 1.58	1.32 ± 1.06	<0.001
	HG&MG	HG=17 MG=19	HG -0.71 vs MG 1.32			<0.001
**Sensitivity analysis (BOCF)**	HG	50	11.94 ± 0.98	11.60 ± 1.18	-0.34 ± 0.82	0.005
	MG	36	9.64 ± 1.27	10.61 ± 1.42	0.97 ± 1.11	<0.001
	HG&MG	HG=50 MG=36	HG -0.34 vs MG 0.97			<0.001

BOCF analyses included participants with available baseline values for the corresponding outcome. Missing post-intervention values were imputed using baseline observation carried forward. Sample sizes varied across outcomes because baseline data were not available for all participants. Blank cells indicate not applicable.

Table S 3. Baseline objective body-composition indicators according to nutritional-status grouping and their correlations with baseline MNA-SF scores

Outcome/Analysis	Variable	HG	MG	Spearman ρ	p value
**A. Baseline comparison of objective body-composition indicators between HG and MG**					
Baseline body-composition analysis	ASM (kg)	16.93 ± 3.88 (n = 57)	15.93±4.45 (n = 63)		0.183
	ASMI (kg/m²)	6.44 ± 0.89 (n = 57)	6.18±1.21 (n = 63)		0.241
	Body fat percentage (%)	32.65 ± 5.29 (n = 57)	31.64±7.14 (n = 63)		0.503
**B. Correlations between baseline MNA-SF scores and objective body-composition indicators**					
HG vs MG	ASM (kg) (n = 80)			0.265	0.018
	ASMI (kg/m²) (n = 80)			0.263	0.018
	Body fat percentage (%) (n = 80)			0.149	0.186

Table S 4. Comparison-specific, taxonomic-rank-stratified Benjamini–Hochberg FDR correction for reported LEfSe-derived taxonomic findings

Comparison	Feature (rank)	Enriched group	Raw p	FDR q
Baseline HG vs MG	*Alloscardovia* (genus)	HG	0.000451	0.000812
	Alloscardovia omnicolens (species)	HG	0.000451	0.000677
	Scardovia (genus)	MG	0.000541	0.000812
	Scardovia wiggsiae (species)	MG	0.000908	0.000908
	Porphyromonas (genus)	MG	0.004534	0.004534
	Porphyromonas sp. HMSC065F10 (species)	MG	0.000244	0.000677
	Porphyromonadaceae (family)	MG	0.002368	0.002368
HG pre vs post	*Paraprevotella* (genus)	Post	0.008216	0.008216
	Paraprevotella xylaniphila (species)	Post	0.008216	0.008216
	Bacteroides stercoris (species)	Post	0.006239	0.006239
MG pre vs post	*Klebsiella* (genus)	Post	0.003795	0.003795
	Klebsiella pneumoniae (species)	Post	0.004569	0.006239
	Klebsiella variicola (species)	Post	0.004405	0.006239
Post-intervention HG vs MG	*Streptococcaceae* (family)	MG	0.003049	0.003049
	Streptococcus (genus)	MG	0.003378	0.003378
	Streptococcus salivarius (species)	MG	0.002477	0.004954
	Streptococcus parasanguinis (species)	MG	0.004110	0.005160
	Streptococcus oralis (species)	MG	0.005160	0.005160
